# Transient facial paresis as a complication of buccal fat removal

**DOI:** 10.1016/j.jpra.2024.09.012

**Published:** 2024-09-19

**Authors:** Alexa Franco, Anna Frants, Manuela von Sneidern, Danielle F. Eytan

**Affiliations:** a NYU Langone Health Department of Otolaryngology-Head and Neck Surgery, New York, NY, USA; b NYU Langone Health, Division of Facial Plastic and Reconstructive Surgery, Department of Otolaryngology-Head and Neck Surgery, New York, NY, USA

**Keywords:** Facial palsy, Facial nerve, Facial nerve palsy, Buccal fat

## Abstract

**Aim:**

This case highlights the rarely reported complication of facial paresis following buccal fat pad removal and its management.

**Background:**

The buccal fat pad is a vital structure in facial aesthetics. In recent years, buccal fat pad removal for mid facial sculpting has gained popularity among patients owing in part to the rise of social media in plastic surgery. Although buccal fat pad removal is usually a safe procedure, potential complications can be quite severe, and can include infection, over-resection, asymmetry, hematoma, facial nerve or parotid duct injury and trismus.

**Case description:**

Herein we describe a case of iatrogenic left facial paresis secondary to buccal fat removal, and discuss the importance of appropriate patient counseling, meticulous technique, and post-operative care in the event of a complication.

**Conclusion:**

High dose corticosteroids and facial therapy can be effective in treating iatrogenic facial palsy secondary to buccal fat pad removal.

**Clinical significance:**

Although buccal fat pad removal has become a common procedure for midface sculpting, the risks remain serious and patient counseling regarding possible complications, including transient facial palsy, is paramount. Meticulous technique as well as knowledge of the relationships between the buccal fat pad, the parotid duct, and the buccal branches of the facial nerve are vital in the prevention of facial paresis when removing buccal fat.

## Background

In recent years, the cosmetic industry and social media have advocated the idea of a slender middle and lower facial profile with accentuated cheekbones as a way to enhance facial beauty. Consequently, there has been a significant rise in the popularity of minimally invasive surgical procedures aimed at achieving this facial aesthetic, including buccal fat pad (BFP) removal.^1^

The BFP is a biconvex, multilobulated encapsulated adipose mass in the middle third of the cheek between the masseter and buccinator muscles that offers support and protection to the vital structures situated in the buccal region, including the branches of the facial nerve and parotid duct.^2^ The buccal branch of the facial nerve originates within the parotid gland as the inferior division of the facial nerve divides at the pes anserinus.^2^ It then splits into multiple unnamed branches which are characterized by their depth, with superficial branches coursing beneath the skin to innervate the superficial muscles of the face, such as the procerus, while the deeper branches pass under and innervate the levator labii superioris and zygomaticus major.^2^^,^^3^ These branches either overlie or lie within the anterior lobe or the buccal extension of the posterior lobe of the buccal fat pad.^2^^,^^3^ The parotid duct and facial vein also pass through the anterior lobe. When accessing the buccal fat pad, it is important to remember that the buccal extension of the posterior lobe lies inferior to the parotid duct and is usually the portion that is removed during aesthetic surgery.^4^ Further avoidance of important structures can be achieved by removing only the fat that either passively protrudes or can be gently teased from the buccal space.^4^

The BFP is also an important anatomical structure in facial aesthetics given it provides contour to the lower face^4^^,5^The partial removal of buccal fat aims to reduce the appearance of excessive cheeks or rounded faces in order to achieve a more angular facial appearance with a high malar region.^1^^,^^4^ This can be achieved via either a facial approach during a facelift procedure or an intraoral approach. Although the intraoral approach is considered safest, both methods can potentially lead to over resection, asymmetry, parotid duct or facial nerve injury, hematoma, trismus and infection.^4^, ^5^, ^6^, ^7^

Herein we describe a case of iatrogenic left facial paresis secondary to buccal fat removal, and discuss the importance of appropriate patient counseling, meticulous technique, and post-operative care in the event of complication.

## Case description

**Figure 1:**
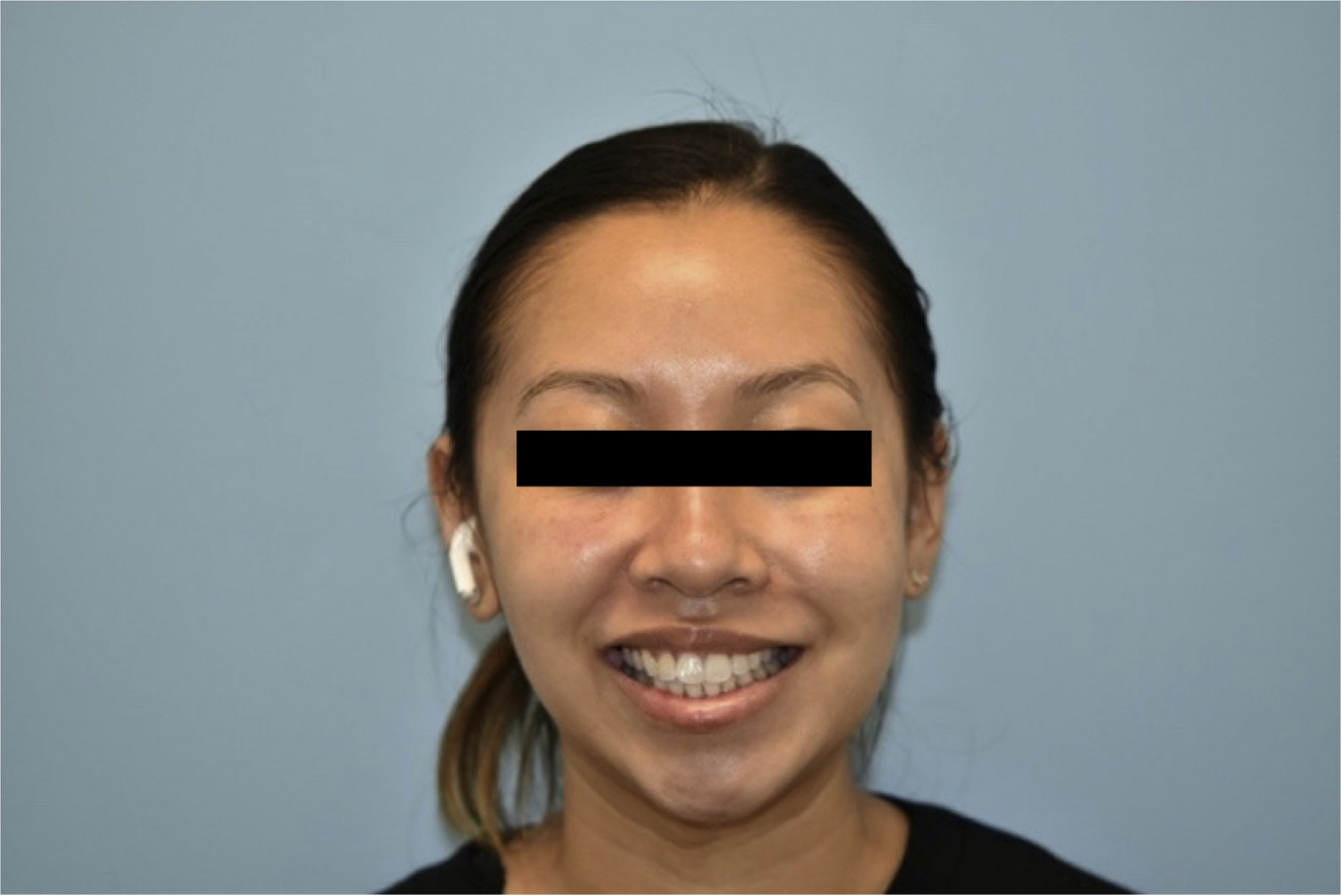
Frontal view with smile three weeks postop.

**Figure 2:**
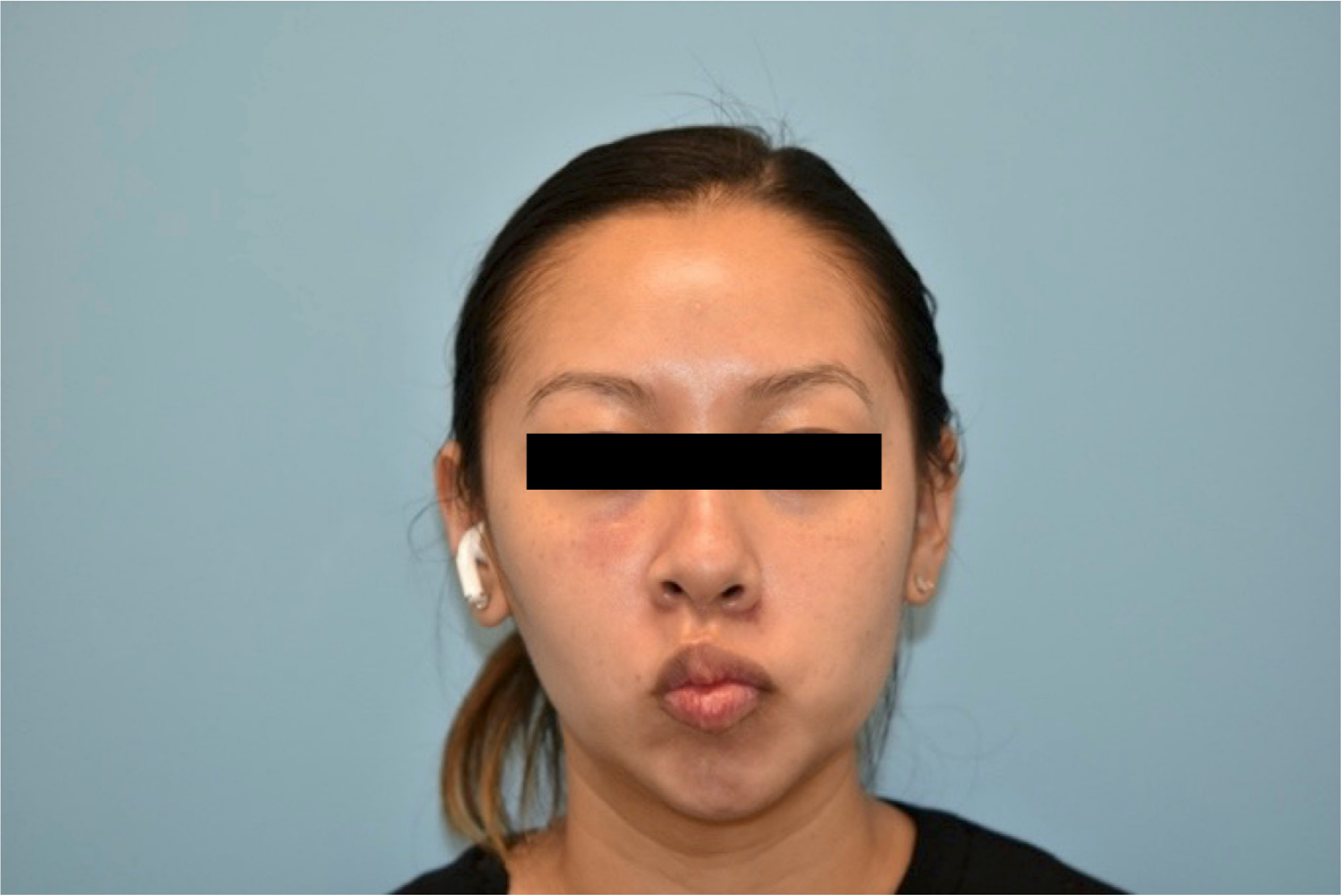
Frontal view with pucker three weeks postop.

**Figure 3:**
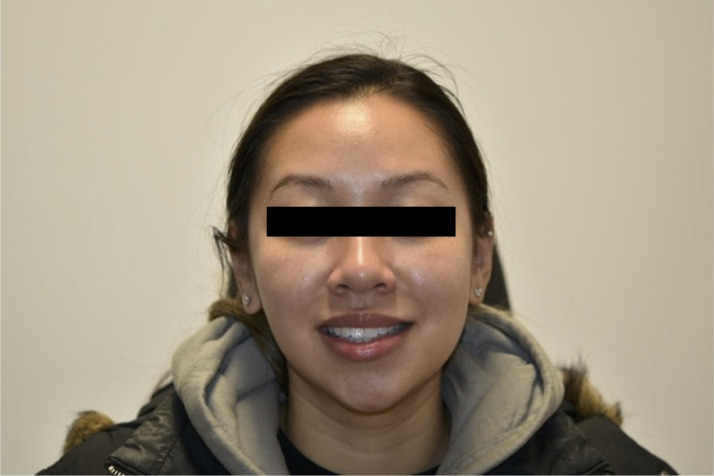
Frontal view with smile three months postop.

**Figure 4:**
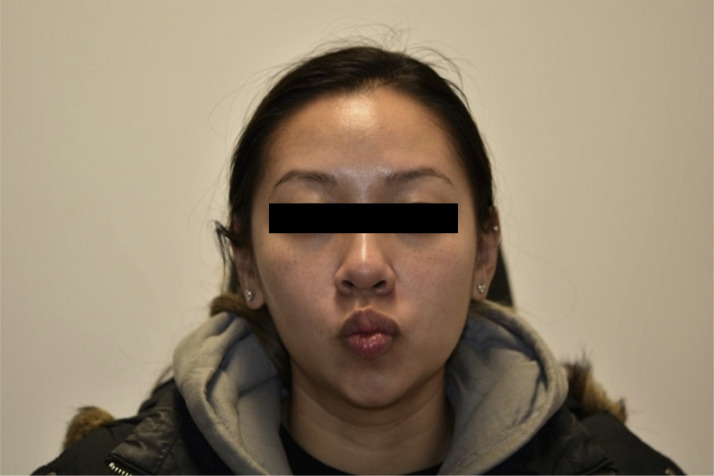
Frontal view with pucker three months postop.

A 32-year-old healthy female presented to our institution for evaluation of left facial paresis after bilateral, intraoral cosmetic buccal fat excision and left nasal ala mole excision by an outside plastic surgeon. In the immediate post operative period she noticed an inability to flare her left nostril as well as drooping of her left upper lip. She also reported mild dribbling from her left oral commissure when drinking thin liquids. The patient later returned to her plastic surgeon who prescribed a Medrol Dosepak containing a 6-day tapering course of methylprednisone beginning at 24 mg for a total of 84 mg. No further workup, including nerve conduction testing, was performed. She completed her steroid course by postoperative day 20 without any appreciable change in facial movement, prompting her to seek care at our institution. On physical exam, there was mild facial weakness present in a buccal distribution on the left with a weak lip pucker and open mouth smile (Sunnybrook score of 76) (Figure 1, Figure 2). All other facial nerve branches were intact and symmetric. She was prescribed an additional 2-week steroid taper beginning at 60 mg of prednisone. The patient was seen again three months postoperatively, at which point she noted mild improvement in movement and tone, particularly at rest and in photos, however still displayed some asymmetry with dynamic movement with a Sunnybrook score of 92 (Figure 3, Figure 4). Facial physical therapy was recommended. She has undergone two sessions of neuromuscular retraining and exercises with subjective improvement in the movement and appearance of her left upper lip, however she continues to endorse minimal resting and dynamic asymmetry.

## Discussion

The BFP, masseter muscle, mandible, and subcutaneous fat all contribute to the contour of the lower face. Therefore, the BFP and its removal have an important role in facial aesthetics.^4^, ^5^, ^6^, ^7^ While not a novel procedure, BFP removal has recently become one of the staples for reducing midface fullness and highlighting the zygomatic prominence.^5^

A meticulous and conservative approach is important to avoid complications, particularly damage to the facial nerve and parotid duct.^4^^,^^7^ Hwang et al. conducted a cadaver study in 2005 to describe the spatial relationships of the buccal branches of the facial nerve, parotid duct, and the BFP.^8^ In 73.7 % of cadavers, they found that the buccal branches of the facial nerve were located superficial to the BFP, whereas in 26.3 % of cadavers these branches traveled through the buccal extension of the BFP. Knowledge of these anatomic variants is paramount in the prevention of facial paresis when removing buccal fat.

Complication rates of buccal fat pad removal have been reported between 0 % and 18 % in the literature.^3^^,^^5^, ^6^, ^7^ Traboulsi-Garet et al. conducted a systematic review to evaluate the efficacy and safety of buccal fat removal and reported seven complications in the 134 procedures identified, including two cases of transient facial paresis.^7^ Despite this low postoperative complication rate, it remains crucial to provide patients with thorough pre-operative counseling regarding the possibility of a transient, albeit adverse cosmetic outcome.

Treatment options to facilitate facial movement recovery after iatrogenic facial nerve injury are varied and include both physical therapy and systemic corticosteroids. Due to the varied and incomplete reporting of facial functional outcomes, previous studies have found it difficult to draw definitive conclusions regarding the effectiveness of physical therapy for treatment of iatrogenic facial paresis.^9^ Conversely, the benefits of high dose systemic steroids for treatment of acute peripheral facial palsy, including iatrogenic facial palsy, have been extensively documented.^10^

## Clinical significance

Although BFP removal has become an increasingly common procedure for midface sculpting, the risks remain serious and patient counseling regarding possible complications, including transient facial palsy, is paramount. Although most iatrogenic facial palsies will recover spontaneously, facial physical therapy provides no harm to patients, and when combined with high dose corticosteroids can be effective in treating iatrogenic facial palsy secondary to buccal fat pad removal.

## Consent

Written informed consent for the anonymized information and images published in this article was obtained from the patient.

## Statement of ethical approval

Ethical approval: not required.

## CRediT authorship contribution statement

**Alexa Franco:** Conceptualization, Investigation, Writing – original draft, Visualization. **Anna Frants:** Conceptualization, Investigation, Writing – original draft. **Manuela von Sneidern:** Conceptualization, Investigation, Writing – original draft, Writing – review & editing. **Danielle F. Eytan:** Writing – review & editing, Supervision, Project administration.

## Declaration of competing interest

The authors have no conflicts of interest to declare.

## References

[1] Cosmetic Surgery National Data Bank Statistics (2017). Aesthet Surg J.

[2] Zhang H.M., Yan Y.P., Qi K.M., Wang J.Q., Liu Z.F. (2002). Anatomical structure of the buccal fat pad and its clinical adaptations. Plast Reconstr Surg.

[3] Dubin B., Jackson I.T., Halim A., Triplett W.W., Ferreira M. (1989). Anatomy of the buccal fat pad and its clinical significance. Plast Reconstr Surg.

[4] Sezgin B., Tatar S., Boge M., Ozmen S., Yavuzer R. (2019). The excision of the buccal fat pad for cheek refinement: Volumetric considerations. Aesthet Surg J.

[5] Moura L.B., Spin J.R., Spin-Neto R., Pereira-Filho V.A (2018). Buccal fat pad removal to improve facial aesthetics: an established technique?. Med Oral Patol Oral Cir Bucal.

[6] Davis B., Serra M. (2022). StatPearls [Internet].

[7] Traboulsi-Garet B., Camps-Font O., Traboulsi-Garet M., Gay-Escoda C. (2021). Buccal fat pad excision for cheek refinement: A systematic review. Med Oral Patol Oral Cir Bucal..

[8] Hwang K., Cho H.J., Battuvshin D., Chung I.H., Hwang S.H. (2005). Interrelated buccal fat pad with facial buccal branches and parotid duct. J Craniofac Surg.

[9] Wamkpah N.S., Jeanpierre L., Lieu J.E.C., Del Toro D., Simon L.E., Chi J.J. (2020). Physical therapy for iatrogenic facial paralysis: A systematic review. JAMA Otolaryngol Head Neck Surg..

[10] Kim S.J., Lee H.Y. (2020). Acute peripheral facial palsy: Recent guidelines and a systematic review of the literature. J Korean Med Sci.

